# Mefloquine-associated dizziness, diplopia, and central serous chorioretinopathy: a case report

**DOI:** 10.1186/s13256-016-1091-4

**Published:** 2016-10-31

**Authors:** Manish Jain, Remington L. Nevin, Iajaz Ahmed

**Affiliations:** 1 Department of Ophthalmology, NMC Specialty Hospital, Al Ain, United Arab Emirates; 2 Department of Environmental Health & Engineering, Johns Hopkins Bloomberg School of Public Health, 615 N. Wolfe St., Baltimore, MD 21205 USA; 3 Department of Medicine, NMC Specialty Hospital, Al Ain, United Arab Emirates

**Keywords:** Central serous chorioretinopathy, Diplopia, Dizziness, Mefloquine, Quinoline, Antimalarial

## Abstract

**Background:**

Many acute and chronic neurological sequelae from the quinoline derivative antimalarial drug mefloquine, including dizziness and effects on the visual system such as diplopia and blurred vision, may be attributable to focal central nervous system toxicity. Maculopathy has also been reported with use of mefloquine, although the mechanism of this effect has remained unclear. Identification of a common mechanism of toxicity plausibly underlying these visual and non-visual effects may provide broader insights into the acute and chronic neuropsychiatric effects of this and other quinoline antimalarial drugs.

**Case presentation:**

This case report describes a 30-year-old man of Pakistani descent with sudden onset of dizziness and diplopia following the administration of mefloquine who developed macular changes diagnosed as acute central serous chorioretinopathy by angiography and optical coherence tomography. Similarities between the visual conditions observed in this case and those observed following administration of related quinoline derivative antimalarial drugs including quinine are considered, and plausible mechanisms for the observed drug-induced toxicity are discussed.

**Conclusions:**

It is proposed that central serous chorioretinopathy be considered a potential ophthalmological sign of mefloquine central nervous system toxicity, and for this effect to potentially indicate susceptibility to other neuropsychiatric effects of mefloquine intoxication. Treating physicians should be aware of the potential for acute and chronic ocular effects resulting from administration of mefloquine and other quinoline antimalarial drugs.

## Background

Mefloquine is a quinoline derivative antimalarial drug structurally related to quinine that has been previously widely used in the treatment and prophylaxis of malaria. Recently its popularity has declined as awareness has grown of the drug’s focal central nervous system (CNS) toxicity, which is associated with a wide range of acute and chronic neurological sequelae including vertigo, loss of balance, and symptoms of polyneuropathy which may be irreversible [1], as well as certain neuropsychiatric effects including cognitive impairment which may last years after use [2]. Although not widely recognized in the literature [3, 4], certain visual effects associated with mefloquine use including blurred vision or accommodative dysfunction may also be plausibly attributable to focal CNS toxicity [1, 5].

While dizziness [1], diplopia [6], and maculopathy [7] have been previously reported separately with mefloquine use, these conditions have not previously been reported together, and confirmation of central serous chorioretinopathy (CSCR) has not been previously reported. Identification of a common mechanism of CNS toxicity plausibly underlying both these visual and non-visual effects would have implications for better understanding of the acute and chronic neuropsychiatric effects of intoxication with mefloquine and other quinoline antimalarial drugs.

We present a case of these adverse events occurring together in a man treated with mefloquine for presumed malaria, and propose the novel theory that CSCR may represent an ophthalmological sign of mefloquine CNS toxicity.

## Case presentation

A 30-year-old man of Pakistani descent was referred to ophthalmology with a history of sudden profound diminution of vision in his right eye 3 days earlier associated with transient diplopia and dizziness. He had a recent history of febrile illness marked by constitutional symptoms including headache and myalgia 20 days earlier when he traveled to his native country. Although no peripheral blood smears or rapid diagnostic testing were obtained, on suspicion of malaria, he was immediately treated by a local physician with 2500 mg of chloroquine over 3 days, followed by 15 mg of primaquine daily over 14 days, and then with 1500 mg of mefloquine in three divided doses over 24 hours. Apart from symptoms related to his initial febrile illness, he was asymptomatic until he received mefloquine. Its introduction was associated with an onset of diplopia, blurred vision in his right eye, dizziness, nausea, and vomiting after intake of the first dose, with blurred vision progressing over the course of dosing. These symptoms prompted the temporary addition of omeprazole and domperidone, and the combination chlordiazepoxide-clidinium bromide, the latter of which was discontinued the day prior to presentation to ophthalmology, by which time all symptoms had resolved but the progressive blurred vision.

There was no history of observed nystagmus nor was there a history of obvious psychiatric symptoms. He smoked tobacco occasionally but did not consume alcohol or recreational drugs. He denied prior use of steroid drugs.

**Fig. 1:**
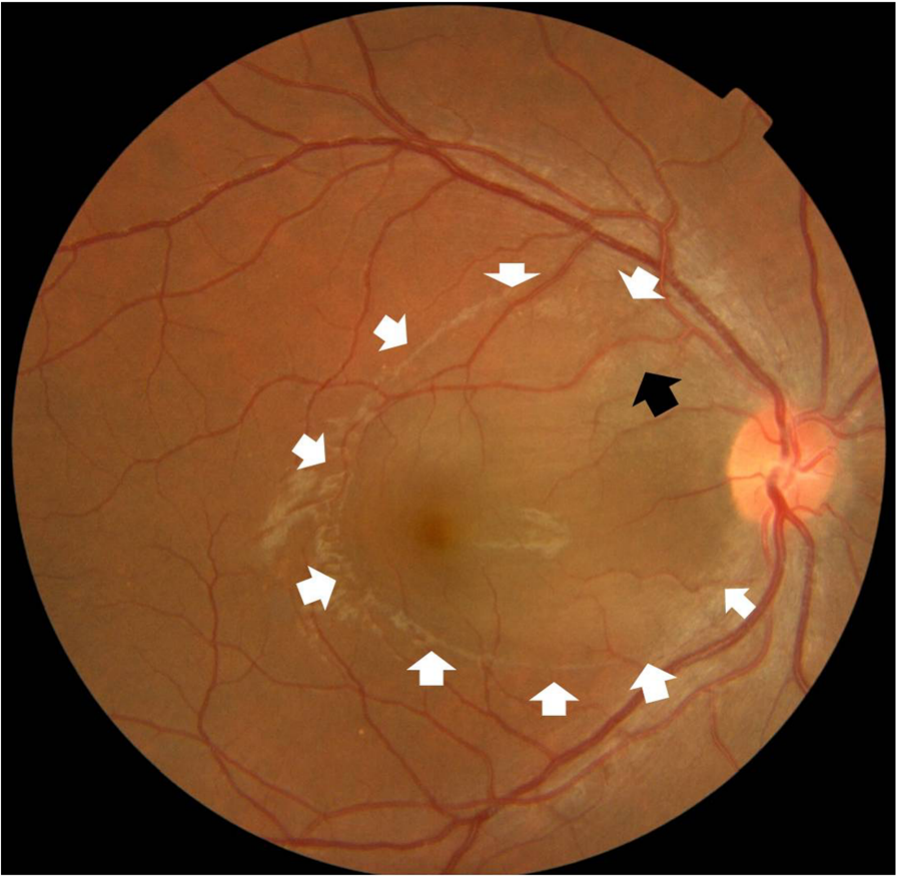
Fundus with site of leak (*black arrow*) showing gross elevation of neurosensory retina (*white arrows*) that includes the entire macula and reaches close to the temporal border of the disc

**Fig. 2:**
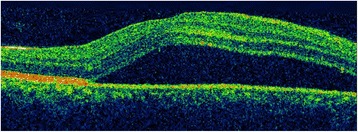
Optical coherence tomography showing the elevation of neurosensory retina, more marked nasally; the central foveal thickness is 453 μm, while the maximum thickness is 794 μm

**Fig. 3:**
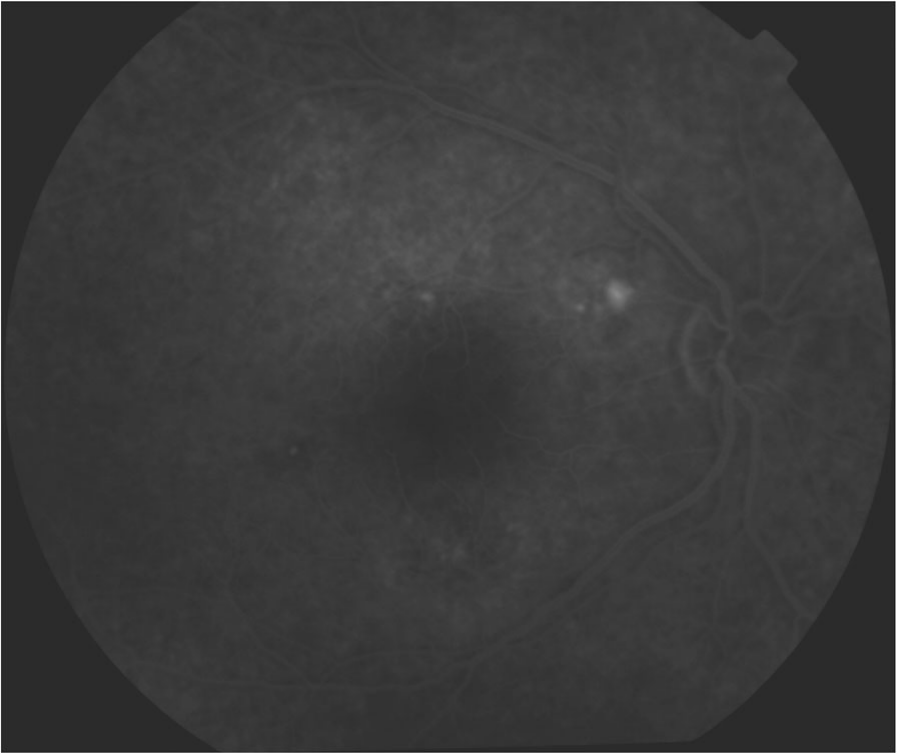
Fluorescein angiography revealing a focal leak that progressed to an ink blot appearance in the late phase

On examination 4 days after the onset of visual symptoms, his uncorrected visual acuity for his right eye (OD; *oculus dexter*) was 20/100 at distance and 20/50 at near, and for his left eye (OS; *oculus sinister*) his uncorrected visual acuity was 20/20 near and distance. His best-corrected visual acuity was unchanged with a manifest refraction of +1.5 diopters (D) sphere OD and +0.25 D sphere OS. Extraocular muscles were unrestricted in both eyes and the cover test demonstrated orthophoria. There was no diplopia. His right eye anterior segment was quiet and had no evidence of cornea verticillata. There was no relative afferent pupillary defect or anisocoria. The intraocular pressure was 18 mm Hg in both eyes. An Amsler grid suggested a large area of central metamorphopsia in his right eye and, corresponding to this, a fundus examination revealed a large ovoid area of dome-shaped serous elevation of retina between the temporal arcades of the retina in his right eye, including the entire macula and reaching close to the temporal border of the disc (Fig. 1). His left eye was normal. His color vision as tested on Ishihara’s tests (38 plates) was normal. Optic coherence tomography (OCT) revealed serous elevation of his right eye neurosensory retina (Fig. 2). Fluorescein angiography revealed focal leaks of the retinal pigment epithelium within one disc diameter superotemporal to the disc that appeared hyperfluorescent in the arterial phase. One of them acquired an ink blot appearance in the late phase (Fig. 3). A diagnosis of unilateral CSCR was made, and he was referred to an internist for further evaluation. His vital signs were normal, and neurological, musculoskeletal, and psychiatric evaluations were noncontributory, although a detailed neuropsychiatric evaluation was not performed. He was asked to discontinue all medicines. His visual acuity steadily improved and the neurosensory retinal elevation receded. Eleven weeks after presentation, he had an uncorrected visual acuity of 20/20 distance and near in both eyes with no abnormality on OCT.

**Fig. 4:**
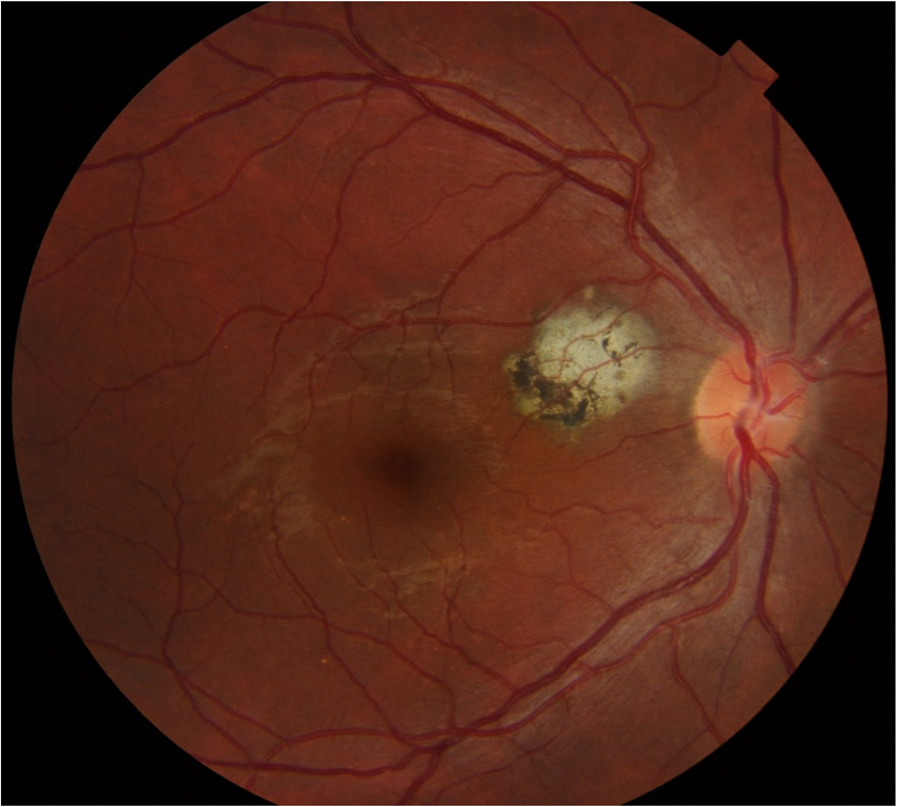
Macular changes 44 months after presentation

Six months later, he reported visual disturbance in his right eye but a clinical examination and OCT ruled out recurrence. However, 1 year after his initial presentation, he reported mild blurring in his right eye. His best-corrected visual acuity was 20/25 distance and 20/20 near, and a leak similar in location to the primary one was noted and treated with focal laser photocoagulation. His visual acuity returned to baseline after 7 weeks. When last seen, 44 months after the initial episode, his visual acuity was stable at 20/20 near and distance in both eyes, and a scar was visible between the macula and the disc representing the area that had focal laser treatment (Fig. 4).

## Discussion

Based on our review of the literature, to the best of our knowledge this is the first report of a confirmed case of unilateral CSCR associated with use of mefloquine. With the exception of our patient’s residual focal scarring, which represents the effects of overly strong focal laser, our patient fortunately made an unremarkable recovery without other chronic sequelae.

Walker and Colleaux suspected a possible bilateral CSCR or inflammatory insult in an asymptomatic patient with macular changes incidentally discovered following from a large cumulative dose (19.5 g) of mefloquine consumed over a year and a half [7]. In contrast, in our patient, CSCR was confirmed in association with transient dizziness and diplopia that developed following acute exposure to the drug.

We consider it unlikely in our case that drugs other than mefloquine could have primarily induced the CSCR and accompanying symptoms. Our patient emphasized that the onset of blurred vision and diplopia occurred within hours of his intake of mefloquine and prior to his use of other drugs used to later manage dizziness and vomiting. He also emphasized that none of these symptoms were observed during administration of earlier antimalarial drugs alone. Although causal attribution to mefloquine is plausible in this case based both on the timing of symptom onset and the drug’s prior reported association with dizziness and diplopia, the underlying mechanism of the CSCR may be less readily apparent.

One possible mechanism for the CSCR could be a direct effect of mefloquine on the retina. Mefloquine and related quinoline antimalarial drugs are potent blockers of connexins, including connexin43 [8], which are expressed throughout the epithelium, glia, and Müller cells, as well as the retinal and choroidal circulation [9] where they serve to facilitate intercellular electrical and metabolic communication through their apposition at cell membranes in hexameric structures known as gap junctions [10]. In this case, it is plausible that connexin43 gap junction blockade at the level of the choroidal vasculature and retinal pigment epithelium could have resulted in increased choroidal vascular permeability and extravasation of fluid underneath the retinal pigment epithelium. For example, gap junction dysfunction in congenital oculodentodigital dysplasia arising from an autosomal dominant mutation of the gap junction protein alpha-1 (*GJA1*) gene coding for connexin43 results in iridociliary cysts, thought to be due to abnormal adhesion of epithelial cells [11]. However, arguing against this possibility in our case is that there were no anterior segment sequelae, implying the absence of similar disruptions elsewhere despite the extensive expression of connexin43 throughout his eye. In addition, such a mechanism does not readily explain the reported dizziness or diplopia, and neither does it explain the unilateral nature of the observed CSCR. In contrast, recent insights into the toxic effects of mefloquine on the CNS [1] may provide an alternative and more plausible explanation for these visual and non-visual effects.

Serous elevation in CSCR is characterized by detachment of the neurosensory retina due to focal leakage of choroidal interstitial fluid. Recent improvements in angiography now suggest that CSCR arises when increased hydrostatic pressure in the choroid associated with choroidal hyperpermeability reverses the normal physiologic flow of fluid from the retina to the choroid [12]. This hyperpermeability may arise with transient capillary and venous hyperemia [13], such as may occur from loss of opposition to sympathetics from decreased parasympathetic tone. Sympathomimetic agents have been associated in the literature with cases of CSCR [14], including unilateral CSCR [15].

Given this pathophysiology, we argue that it is plausible that the unilateral CSCR in this case could be attributable to a transient loss of parasympathetic innervation to the ipsilateral ciliary ganglion (CG), leading to alterations in choroidal blood flow. Direct focal effects on either the ipsilateral parasympathetic preganglionic neurons in the Edinger–Westphal nucleus (EWN) [16], which relay in the CG to postganglionic neurons innervating both the iris and smooth muscle regulating choroid blood flow [17, 18], or on afferents in the hypothalamus supplying the EWN, including the suprachiasmatic nucleus (SCN) [18], could conceivably cause these effects.

Mefloquine has been previously shown to directly affect electrical activity in the hypothalamus [19], and histopathologically confirmed focal neurotoxic lesions within the hypothalamus [20, 21] and in the vicinity of the EWN [22] have been previously reported with related quinoline derivative antimalarial drugs.

Other symptoms consistent with loss of CG parasympathetic innervation, including loss of accommodation and mydriasis [23] responsive to local pilocarpine [24], phenylephrine [25], or to stellate ganglion block [25] and associated with sudden visual disturbance, including unilateral blindness [26], have been previously reported from the related quinoline antimalarial quinine.

Consistent with other confirmed focal CNS effects of mefloquine, including on the brainstem vestibular [27], gracilis, and cuneatus nuclei [28], a reversible transient effect of mefloquine on the functioning either of the ipsilateral EWN, or upstream on hypothalamic centers, would provide a highly parsimonious explanation for the observed unilateral CSCR. In this case, the persistent visual disturbance following resolution of acute symptoms may be considered secondary to the resultant serous elevation of the neurosensory retina causing retinal dysfunction, which would resolve only over a prolonged period.

Correspondingly, a reversible transient effect on adjacent oculogyric centers might similarly explain our patient’s reported brief diplopia, while a similar transient effect on adjacent vestibulo-oculomotor centers might explain his brief dizziness [27]. Transient blurring of vision with mefloquine, consistent with a reversible anomalous accommodation, has been previously reported [29], and is a feature of idiosyncratic quinoline toxicity, including from quinine [30] and from numerous experimental quinoline antimalarial drugs [31–33]. Lack of observed pupillary abnormality and diplopia at our patient’s initial presentation would be consistent with such transient effects resolving early.

With regard to the plausible effect of other drugs, either a retinal or CNS mechanism could provide for the concomitant drugs reported in this case contributing to a complex synergistic toxicity. Both chloroquine and primaquine would be expected to modulate the P-glycoprotein (P-gp)-mediated efflux of mefloquine [34], either within the retinal pigment epithelium or within the CNS, potentially contributing to its abnormal accumulation. Similarly, domperidone [35], chlordiazepoxide [36], and omeprazole [37] are known substrates of P-gp, and any of these drugs may have further exacerbated mefloquine’s effects by competing for available drug transport sites in the blood–retinal or blood–brain barriers.

## Conclusions

In this case, principally on the basis of parsimony, we postulate that mefloquine — either alone or in synergy with other P-gp substrates or quinoline antimalarial drugs — caused dizziness, diplopia, and CSCR through transient focal effects on specific structures of our patient’s CNS. This case raises questions as to the possible existence of a CNS class effect among closely related quinoline antimalarial drugs including quinine, which have been previously associated with similar transient unilateral blindness and blurring of vision.

We propose that CSCR should therefore be considered a potential sign of mefloquine intoxication [1] and more generally of quinoline CNS toxicity, and should prompt a careful clinical evaluation for other evidence of neuropsychiatric toxicity. In our case, insight into a possible CNS etiology came too late to inform clinical application, but fortunately our patient appears to have been spared the chronic cognitive sequelae, including cognitive dysfunction, which may affect a sizable minority of those reporting neuropsychiatric adverse effects from the drug [2].

Treating physicians should refrain from using mefloquine in patients with a history of CSCR and should be aware of the potential for acute and chronic ocular effects resulting from the drug’s administration, particularly in combination with other P-gp substrates or inhibitors. During prophylactic use, the onset of such effects should be considered potential evidence of idiosyncratic susceptibility to mefloquine intoxication, and should prompt the immediate substitution of the drug for an alternative non-quinoline antimalarial.

## Abbreviations

CG: Ciliary ganglion
CNS: Central nervous system
CSCR: Central serous chorioretinopathy
D: Diopters
EWN: Edinger–Westphal nucleus
*GJA1*: Gap junction protein alpha-1
OCT: Optic coherence tomography
OD: Right eye (*oculus dexter*)
OS: Left eye (*oculus sinister*)
P-gp: P-glycoprotein
SCN: Suprachiasmatic nucleus
